# Burden of Complicated Malaria in a Densely Forested Bastar Region of Chhattisgarh State (Central India)

**DOI:** 10.1371/journal.pone.0115266

**Published:** 2014-12-22

**Authors:** Vidhan Jain, Sanjay Basak, Sneha Bhandari, Praveen K. Bharti, Trilok Thomas, Mrigendra P. Singh, Neeru Singh

**Affiliations:** 1 Regional Medical Research Centre for Tribals (RMRCT), ICMR, Garha, Jabalpur, Madhya Pradesh, India; 2 District Malaria Office, Maharani Hospital and associated Medical College Jagdalpur, Chhattisgarh, India; 3 National Institute of Malaria Research, Field Unit, Jabalpur, Madhya Pradesh, India; Centro de Pesquisa Rene Rachou/Fundação Oswaldo Cruz (Fiocruz-Minas), Brazil

## Abstract

**Background:**

A prospective study on severe and complicated malaria was undertaken in the tribal dominated area of Bastar division, Chhattisgarh (CG), Central India, with an objective to understand the clinical epidemiology of complicated malaria in patients attending at a referral hospital.

**Methods:**

Blood smears, collected from the general medicine and pediatric wards of a government tertiary health care facility located in Jagdalpur, CG, were microscopically examined for malaria parasite from July 2010 to December 2013. The *Plasmodium falciparum* positive malaria cases who met enrollment criteria and provided written informed consent were enrolled under different malaria categories following WHO guidelines. PCR was performed to reconfirm the presence of *P.falciparum* mono infection among enrolled cases. Univariate and multivariate logistic regression analysis was done to identify different risk factors using STATA 11.0.

**Results:**

A total of 40,924 cases were screened for malaria. The prevalence of malaria and *P.falciparum* associated complicated malaria (severe and cerebral both) in the hospital was 6% and 0.81%, respectively. *P.falciparum* malaria prevalence, severity and associated mortality in this region peaked at the age of>4–5 years and declined with increasing age. *P.falciparum* malaria was significantly more prevalent in children than adults (P<0.00001). Among adults, males had significantly more *P.falciparum* malaria than females (P<0.00001). Case fatality rate due to cerebral malaria and severe malaria was, respectively, 32% and 9% among PCR confirmed mono *P.falciparum* cases. Coma was the only independent predictor of mortality in multivariate regression analysis. Mortality was significantly associated with multi-organ complication score (P = 0.0003).

**Conclusion:**

This study has revealed that the pattern of morbidity and mortality in this part of India is very different from earlier reported studies from India. We find that the peak morbidity and mortality in younger children regardless of seasonality. This suggests that this age group needs special care for control and clinical management.

## Introduction

Malaria is a global public health problem and India contributes substantially to global malaria incidence [1]. Factors influencing malaria in India are highly diverse and vary within country as well as from other malaria endemic countries. *Plasmodium falciparum* infection causes the most serious form of malaria and its presentation varies from one place to another depending on factors such as the presence of vectors, parasite species, drug resistance, socioeconomic factors, geographical areas and type of intervention measures used [2].

In India, major studies conducted so far on severe and complicated malaria are from Rourkela and Cuttack, Orissa, East India [3], [4], [5], Bikaner, Rajasthan, Western India [6] and Jabalpur, Central India [7]. All of these studies have been carried out in tertiary level hospitals with very different malaria endemicity and ecosystems.

In an outbreak investigation conducted in Rajasthan, a desert zone, and a low endemic area for malaria, the mortality (34%) was found due to cerebral malaria (CM) in older children and adults (>14–74 years) [6]. Rourkela, an industrial township, is highly endemic for malaria and the mortality was 23% [4] also in older children and adults (>12 years). However, a study carried out earlier by the same group in the same hospital showed equal prevalence of CM in children (<15 years) and adults with high mortality in adults (90%) [3]. In Cuttack, another highly endemic area, studies conducted on pediatric age group found the case fatality rate (CFR) of 12%, [5]. While Jabalpur, a meso-endemic area, showed mortality up to 21% in all age groups including young children [7]. In all of these studies, *P. vivax* and *P. falciparum* were found as mono infections and these studies did not clearly define the clinical picture of CM from other severe forms of malaria (SM). Moreover, none of these studies used PCR to confirm mono-infection of *P. falciparum*.

The present study was carried out in Chhattisgarh (CG), a highly malarious state contributing 12% of malaria and the highest proportion of deaths due to malaria (17%) in the country [8]. All four species of malaria parasites are found in CG i.e. *P. vivax*, *P. falciparum*, *P. ovale* and *P. malariae* [9]. This prospective study was undertaken with the objective to understand the clinical epidemiology of CM and SM. To the best of our knowledge, this is the first study describing the burden of CM/SM in a remote tertiary health facility in a tribal district of India, where all four parasite species are found. The findings from this study will provide evidence, which can be used for development of region-specific malaria control and elimination strategies in this area.

## Patients and Methods

### Study site

**Figure 1:**
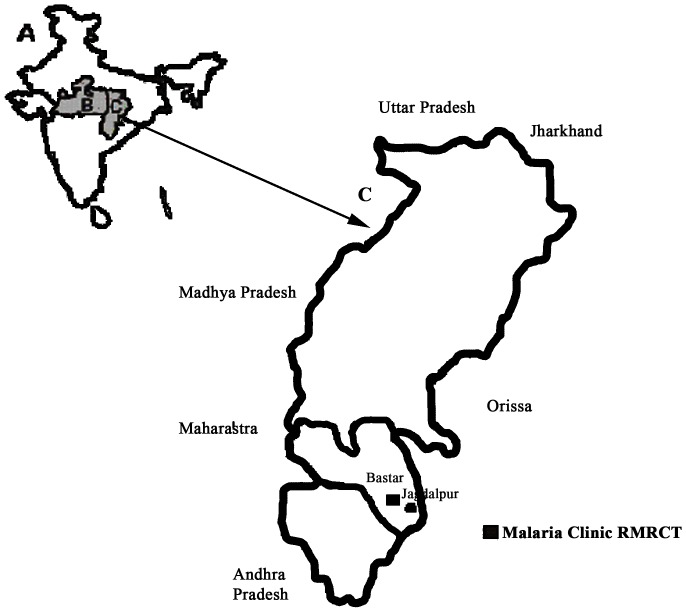
Map of study site. The map shows India (A), state of Madhya Pradesh, where RMRCT is located in Jabalpur (B), state of Chhattisgarh (C). Malaria clinic of RMRCT at Maharani Hospital and associated Medical college Jagdalpur is shows in Bastar region in Chhattisgarh.

A malaria clinic of the Regional Medical Research Centre for Tribal (RMRCT) was established in a 600 bedded Maharani Hospital, which is part of the Medical College in Jagdalpur, CG, India (Coordinates 19.07°N and 82.03°E) in the year 2010 (Fig. 1, A–C). This is the only tertiary health care facility in Bastar division, for seven districts i.e., Kanker, Kondagaon, Jagdalpur, Dantewada, Bijapur, Narayanpur and Sukma. This tertiary health care centre is situated in a remote area of Bastar having thick dense forest (45% of the geographical area under forest) and tribal settlement (75% tribal population). South Bastar of CG is a high-prevalence malaria region, which contributed 24% (30,193/124,006) malaria and 27% (24/90) malaria related deaths in the state in 2012. The Annual Parasite Incidence (API) was 17.5 (*P. falciparum* percentage of 85% (NVBDCP, unpublished report) and Slide Positivity Rate (SPR) of 9%. Average annual rainfall in this region is around 1400–1500 mm. The economy of the villagers residing in this tribal district is mainly agricultural and forest based.

Prior studies carried in CG in 1975–76 revealed, *P. vivax* was the predominant species (58%); however, since 1979 *P. falciparum* showed a steady upward trend (50%) and in 2002, *P. vivax* came down to 28% [10], [11]. Recently in 2012, the API for CG was 5 and SPR and Slide falciparum rate (SFR) of 3.3 and 2.6%, respectively. The *P. falciparum* percentage was 78.2. Among the known vectors are *An. culicifacies*, *An. fluviatilis*, *An. subpictus,* and *An. varuna* [12], [13]. The main tools of malaria control are rapid diagnostic tests (RDTs), ACTs (artemisinin based combination therapy) along with two rounds of alpha-cypermethrin (5%) for indoor residual spray. Further, long lasting insecticide treated bednets (LLINs) were also distributed under the program from the year 2011 onwards (state, VBDCP office, CG).

### Ethical statement

The study protocol was approved by the Institutional Ethical Committee (IEC) of RMRCT, Indian Council of Medical Research (ICMR), New Delhi. Written informed consents were obtained prior to enrollment of the patients, just after the initiation of the treatment. The consent form was provided and explained in detail to the patient either directly (if the subject was conscious, oriented and aged ≥15 years) or to their parents or guardians (if the subject was conscious, oriented and aged up to14 years i.e., children). For unconscious and disoriented patients of all the age groups, consent has been obtained from parents or guardians of the participant. All the consent forms were also witnessed and signed by one more representative of the patient. This clinical investigation has been conducted according to the principles expressed in the declaration of Helsinki.

### Study procedure

This prospective study was carried out from July 2010 to December 2013. All clinically suspected individuals admitted in the general medicine and pediatric wards were screened for malaria parasite by microscopy. RDTs were used only in case of emergency and also when blood smears were doubtful in patients presenting semiconscious/unconsciousness or treated before in case of referral to confirm parasitological diagnosis. Microscopically confirmed *P. falciparum* positive malaria subjects were classified under different categories as per the clinical and laboratory characteristics i.e., mild malaria (MM), severe malaria (SM) and cerebral malaria (CM) following WHO guidelines [7] [14], [15]. SM (cerebral signs were absent in patients) and CM cases were collectively referred as complicated malaria. Based on the treatment outcomes, SM cases were further classified as severe malaria survivors (SM-S) and non survivors (SM-NS). Similarly, CM patients who survived were classified as cerebral malaria survivors (CM-S) and those who died as cerebral malaria non survivors (CM-NS).

### Recognition of complicated malaria

Severe and complicated forms of malaria were recognized by the following definitions. Cerebral malaria (CM) – *P. falciparum* associated unarousable coma which persisted at least for 6 hours (Glasgow coma score ≤10) or ≥3 generalised convulsions in the last 24 hours after correction of hypoglycemia (blood glucose <40 mg/dl) as per the standard guidelines [15]. Other encephalopathies were ruled out by appropriate general clinical examinations (cerebrospinal fluid examination was not done).

Severe malaria (SM) - Presence of any of the following complications in absence of cerebral involvement; acute renal failure - serum creatinine>3 mg/dl or insufficient urine output in adults/children, severe malaria anemia (SMA) - hemoglobin <5 g/dl, jaundice - serum bilirubin>3 mg/dl (jaundice alone was not considered as SM during analysis), hypoglycemia - blood glucose <40 mg/dl, hypotension - systolic blood pressure <80 mm Hg for adults and <50 mm Hg for children (<5 years), respiratory distress – laboured breathing signed by chest/intercostals muscles recession or age stratified increased respiratory rate (>32/minute for adults,>40 in children 5 to 14 years,>50 in children less than 5 years and hyperparasitemia – finger prick peripheral blood smear parasitemia>5% (hyperparasitemia alone was not considered as SM criteria during analysis) according to World Health Organization (WHO) definition [15], [16]. Multi-organ dysfunction syndrome (MODS) was defined by the presence of two or more complications of CM, shock, renal failure, respiratory distress, jaundice, severe malaria anemia, hypoglycaemia, hemolysis/bleeding, and hyperparasitemia. Each of the above complication was assigned one mark and multi organ complication score of a subject was derived by addition of numbers of complications.

Patients presenting none of all the above signs were defined as MM. Clinical and demographic details of subjects were extracted from the hospital records in a structured abstraction form.

### Treatment

All *P. falciparum* malaria patients were treated with body weight dependent doses of intravenous quinine/artesunate followed by oral quinine/artesunate – sulfadoxine and pyrimethamine tablets as per the guidelines of the National Vector Borne Disease Control Programme, Ministry of Health and Family Welfare [17]. In supportive treatment, intravenous fluids and antibiotics were also given. Convulsions were maintained by body weight dependent doses of diazepam/phenytoin. Blood transfusions were given to the anemic patients immediately as per the advice of the consultant/resident doctor after hospitalization. Transfusions were also given to the patients who became considerably anemic during the course of treatment. Patients under respiratory distress were immediately given oxygen inhalation and nabulization. Severe and complicated cases were immediately catheterized by the hospital staff to check urine output and hematurea. Those detected acute renal failure, were treated with diuretic medicines and vesopressors. Severely ill patients were referred to a government specialty tertiary hospital in the state capital Raipur about 300 kilometres (km) North of Jagdalpur (these cases have been followed-up for final outcome).

### Sample collection and processing

Few drops of the finger-prick blood was obtained, for the blood smear preparation (thick and thin) on a glass slide, estimation of hemoglobin (Hb: measured by Hemocue cuvettes, HemoCueAB, Angelholm, Sweden) and random blood sugar (measured by commercially available strips, Contour TS- Bayer CropScience Ltd, India). Filter paper blood spots were taken for PCR diagnosis.

Blood smears were stained by Jaswant Singh and Bhattacharyaji stain [18] and observed under 100× objective using immersion oil (1000 times magnification). Follow-ups of the cases were done on a 12 hour basis for the first 24 hours and further after 48 hours of treatment and also at discharge from the hospital. Percent peripheral parasitemia has been derived after counting the number of parasitized red blood cells (PRBCs) in 1,800 RBCs in an evenly spread area of a thin smear [19].

### Diagnostic Polymerase Chain Reaction (PCR)

PCR was performed to rule out mixed infections among enrolled cases for final clinical analysis. Blood spotted area was punched and put into a 1.5 ml tube. The blood spots were soaked in 150 µL TE buffer (10 mM Tris, 0.1 mM EDTA, pH 8.0) and incubated for an hour at room temperature. After one hour incubation, tubes were placed in a dry bath at 50°C and incubated for 15 minutes and punched by pipettes tips several times. Finally the tubes were incubated at 97°C for 15 minutes and centrifuged at 8,000 rpm for 2 minutes. Supernatant was aspirated and stored at −20°C for PCR amplification. Species-specific nested PCR was carried out for the detection of *P. falciparum* (Pf), *P. vivax* (Pv), *P. malariae* (Pm) and *P. ovale* (Po) using the 18s rRNA gene [9], [20]. Species-specific nested PCR for *P. knowlesi* was also carried out [21].

### Data analysis

Categorical variables were summarized as frequency (proportion). Chi-square or Fisher exact tests were used for comparison of categorical data (clinical complication pattern in adults/children) and prevalence's of malaria, *P. falciparum* malaria, complicated malaria and associated mortality between age groups (adults & children) and gender (male & females) as end points. Univariate and multivariate logistic regression analyses were performed for risk factor analysis with background, demographic and clinical complications as independent variable and complicated malaria and mortality as dependent variables. Prior to the regression analysis presence or absence of the risk factors under independent variables and outcomes in the form of complicated malaria (Yes/No) and mortality (Yes/No) under dependent variables were coded as 1 (Yes) or 0 (No). Variables which showed significance at the level of *p* ∼ 0.1, in univariate analysis were selected and analyzed in multivariate logistic regression to reveal their independent association with disease severity/outcome.

Continuous variables were presented as median and 25^th^ – 75^th^ percentile distribution (IQR). These were tested for normality and analyzed by multiple comparisons ANOVA. If found normally distributed among all study groups (haemoglobin, temperature). In case where the data deviated from the normal distribution, Kruskal-Wallis test was applied (history of illness, duration of hospital stay, blood urea, serum creatinine, systolic blood pressure, respiratory rate, blood glucose, parasitemia, serum bilirubin and pulse temperature). Statistical analysis was performed using STATA version 11.0 software (StataCorp, College Station, TX, USA). Non parametric spearman correlation coefficient was used to determine the correlation between variables (mortality and multi organ complication score). Statistical significance was assumed if a null hypothesis could be rejected at p = 0.05. General malariometric characteristics were defined as follows: Malaria prevalence  =  Total number of positive cases in the hospital ×100/Total number of admitted cases; Case fatality rate (CFR)  =  in a period of time, number of cases died due to specific disease in a hospital ×100/Total number of cases diagnosed with the same disease; *P. falciparum (Pf)%*  =  Total number of *P. falciparum* cases ×100/Total number of cases positive for malaria parasite.

## Results

### Screening of malaria

A total of 40,924 cases in all the age groups with and without fever were microscopically screened for malaria. Of the 2,450 malaria positive cases, 2,099 cases were *P. falciparum,* 340 cases were *P. vivax* and 11 cases were found to be mixed infections of both. Overall malaria prevalence in the hospital was 6% (2,450/40,924) and Pf% was 86.1(2,110/2,450). Among *P. falciparum* cases gametocyte carriage rate was 1.9% (40/2,099).

**Figure 2:**
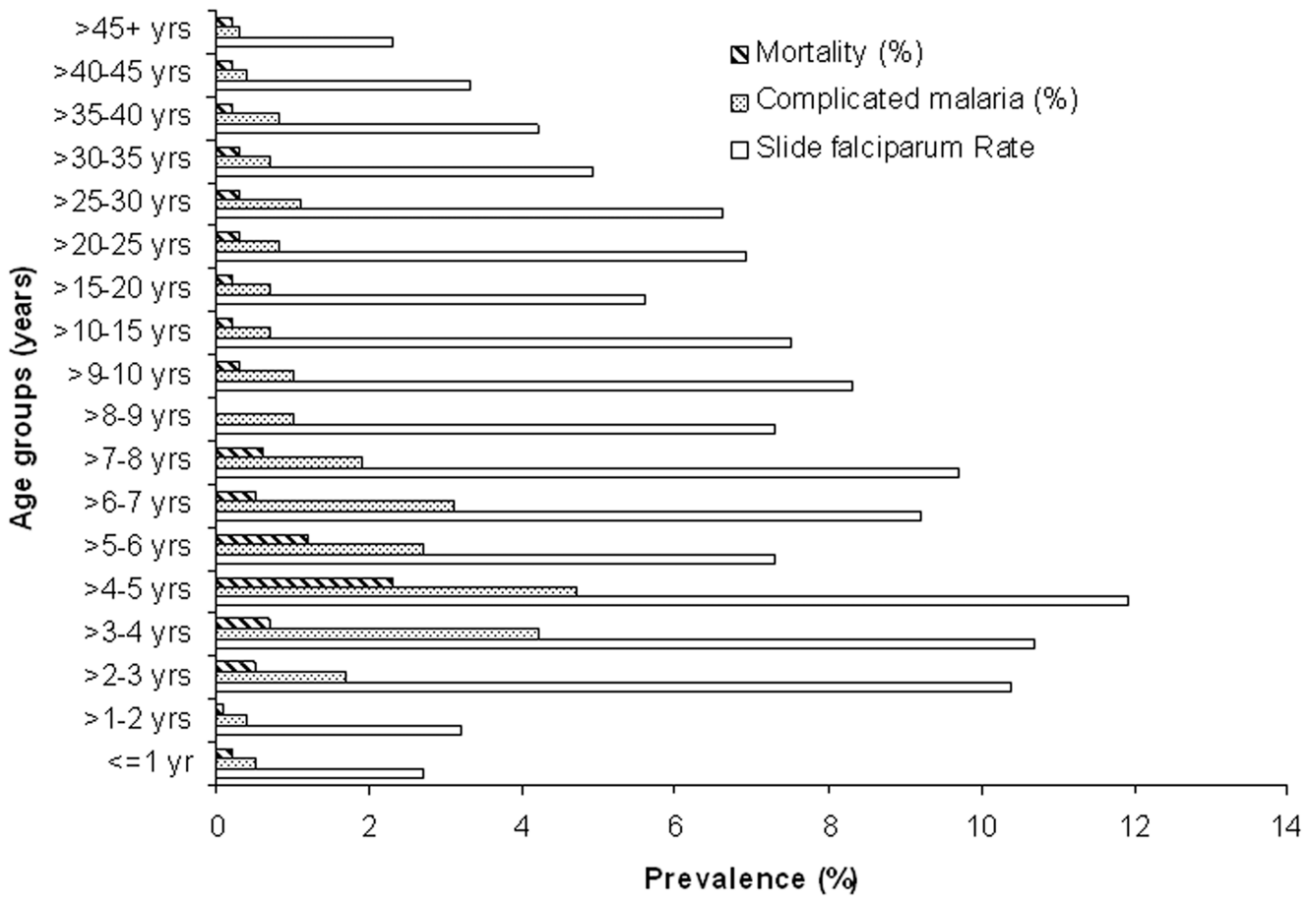
Age wise *P. falciparum* malaria, complicated malaria and associated mortality in a tertiary care hospital in Bastar, Chhattisgarh, Central India. The age group interval is kept one year for children up to 10 years of age. For older children and adults age group interval is five years.

Further age group wise (<15 years & ≥15 years) and sex wise analysis revealed that malaria and *P. falciparum* malaria in particular, was significantly more associated with children [7.9% (689/8,685) & 7.0% (610/8,685)] than adults [5.5% (1,761/32,218) & 4.7% (1,500/32,218)] (χ^2^ = 74.0; *p*<0.00001 and χ^2^ = 78.4; *p*<0.00001 respectively). Out of the 2,110 *P. falciparum* (including mixed *Pf*+*Pv*) cases, 334 had complicated malaria, of which 330 were due to *P. falciparum*, 1 severe *P. vivax* and 3 cases were due to mixed (*Pf*+*Pv*) infections (Table 1). Males were likely to be more at risk of getting malaria and *P. falciparum* infection than females (p<0.00001). An increase in malaria associated morbidity and mortality from the age <1 year to 5 years was observed followed by a decline up to the age group>45 years (Fig. 2).

**Table 1:** Age and sex wise prevalence of malaria among suspected malaria cases at tertiary health care hospital in Bastar, Chhattisgarh, Central India.

Age Group	Sex/Age Group	Screened	+ve	Pf	Pv	Mix(Pf+Pv)	Complicated Malaria (CM+SM)	Mortality	SPR	SFR	Pf%	%Mortality
>15 Years (Adults)	Male	18975	1329	1117	208	4	160†	55	7.0	5.9	84.3	0.3
	Female	13243	432	377	53	2	56	19	3.3	2.9	87.7	0.1
< = 15 Years (Children)	Male	4938	406	352	51	3	69‡	21	8.2	7.2	87.4	0.4
	Female	3747	283	253	28	2	49	14	7.6	6.8	90.1	0.4
Total (A)	Male	23924	1735	1469	259	7	229††	76	7.3	6.2	85.1	0.3
	Female	17000	715	630	81	4	105	33	4.2	3.7	88.7	0.2
Total (B)	Adults	32218	1761	1494	261	6	216‡‡	74	5.5	4.7	85.2	0.2
	Children	8685	689	605	79	5	118¥	35	7.9	7.0	88.5	0.4
Grand Total		40903§	2450	2099	340	11	334#	109	6.0	5.2	86.1	0.3

§ age of 21 cases is missing.

† Having 1 mixed, 1 P. vivax.

‡ Having 2 mixed.

†† Having 3 mixed, 1 P. vivax.

‡‡ Having 1 mixed, 1 P. vivax.

¥ Having 2 mixed.

# Having 3 mixed, 1 P. vivax.

+ve: Positive for malaria; Pf: *Plasmodium falciparum*; Pv: *Plasmodium vivax*; Mixed: *P. falciparum* + *P. vivax*; SPR: Slide Positivity rate; SFR: Slide Falciparum Rate; Pf%: *P. falciparum* percentage; Complicated malaria: Cerebral and Severe malaria both); CM: Cerebral malaria; SM: Severe malaria.

Out of 333 complicated cases of *P. falciparum* malaria, 238 had CM of which 90 died (CFR = 37.8%) and 95 had SM of which 18 died (CFR = 18.9%) which is significant statistically (p = 0.001). We observed complicated malaria cases throughout the year. However, malaria prevalence was significantly higher in the monsoon season (χ^2^ = 106.2, *p*<0.00001) and post monsoon season (χ ^2^ = 166.4, *p*<0.00001) as compared to the dry season.

### Enrollment

A total of 401 microscopically positive *P. falciparum* mono-infection cases out of 2,099 were enrolled and categorized into different disease categories (MM = 121, SM = 95, CM = 185). Out of 401 cases, fourteen cases were found to be infected with mixed *Plasmodium* infection by PCR and were excluded from the analysis (8 CM, 4 SM and 2 MM). Of the remaining 387 cases, thirteen SM were further excluded as they had only jaundice and another 22 SM were excluded due to the presence of only hyperparasitemia as a severe complication. One CM was excluded due to ambiguity in the results.

Finally we compared 119 MM with 232 complicated malaria patients (SM = 56 and CM = 176) to identify background and clinical risk factors for complicated malaria, and age group wise clinical complication patterns and prognosticators. CFR due to CM and SM among PCR confirmed mono *P. falciparum* was 32.4% (57/176) and 8.9% (5/56) respectively, which was found significant (p = 0.001).

Primary complaints among enrolled subjects were, fever with chills and rigor in 90.5%, headache in 79.7%, vomiting in 63.9% and diarrhea in 20.9% cases. Anemia (Hemoglobin <11 mg %) was recorded in 46% cases.

### Continuous clinical variables & complicated malaria

Details of continuous variables investigated in this study are shown in Table 2. Glasgow coma score in CM-NS group was significantly lower than CM-S (*p* = 0.0082). Overall, mortality was significantly higher among comatose patients compared to non-comatose patients (χ^2^  =  11.0, *p* = 0.0009). Median fever history of different groups was similar (*p* = 0.296). Duration (hours) of coma among 23 CM-NS cases and 43 of CM-S patients before hospitalization was not significantly different (*p* = 0.08) [duration in CM-NS group was 12, IQR (8–16) hours than CM-S group 9 IQR (6–11) hours].

**Table 2:** Malaria category-wise clinical characteristics of enrolled subjects.

S No.	Variables	MM (n = 119)	SM-S (n = 49)	CM-S (n = 111)	CM-NS +SM-NS (n = 62^#^)	*P* value
1.	Days of illnesses: Median (IQR)	4 (2–6)	4 (3–6)	4 (3–5)	4 (3–5)	0.296
2.	Percent peripheral parasitemia: Median (IQR)	0.8 (0.4–1.9)	4.8 (1.1–13.6)	1.7 (0.3–6.6)	1.9 (0.5–12.3)	0.0001
3.	Respiratory rate (per minute): Median (IQR)	20 (18–24)	27 (24–31)	26 (24–30)	28 (24–37)	0.0001
4.	Pulse (per minute): Median (IQR)	87 (80–100)	97 (82.5–114.5)	91 (80–110)	103 (83–116)	0.0027
5.	Temperature (^0^F): Median (IQR)	99.2 (98.6–101)	99 (98–99.8)	99.2 (98.2 – 100.0)	99 (98–101.1)	0.176
6.	Systolic BP, (mm Hg): Median (IQR)	110 (104–117)	95 (80–110)	110 (100–120)	124 (107–134)	0.0001
7.	Hemoglobin (g/dl): Median (IQR)	11.5 (10.0–13.6)	9.3 (7.2–11.4)	10.7 (7.9–12)	9.7 (8.4–11.9)	<0.0001
8.	Total leukocyte count/mm^3^: Median (IQR)	5750 (3900–7600)	8500 (6850–9900)	9050 (7600–9800)	8850 (7200–14400)	0.0001
9	Blood glucose (mg%): Median (IQR)	120.5 (102–144.5)	109.5 (88.5–139.5)	144 (109–193)	130 (95–222)	0.0001
10.	Serum bilirubin (mg%): Median (IQR)	1.6 (1.3–1.9)	7.6 (4.4–10.0)	4.7 (2.5–7.2)	5.5 (2.9–15.4)	0.0001
11.	Serum creatinine (mg%): Median (IQR)	1.4 (0.9–1.8)	1.4 (0.8–1.7)	1.6 (1–3.4)	2 (1.2–3.3)	0.191
12.	Blood urea (mg%): Median (IQR)	33 (26–86)	70 (50–87)	78 (38–174)	117 (44–192.5)	0.068
13	MCS, (IQR): Median (IQR)	0	2 (2–3)	2 (2–3)	3 (2–4)	0.0002^*^
14	Glasgow coma score (3–14): Median (IQR)	14 (14–14)	14 (13–14)	7 (6–9)	6 (3–8)	0.0082^**^

MM  =  Mild malaria, SM-S  =  severe malaria survivors, CM-S  =  cerebral malaria survivors, CM-NS^#^ =  cerebral malaria non-survivors (57 were CM-NS and 5 were SM-NS (severe malaria non-survivors); Severe malaria (SM) cases had no cerebral signs. MCS  =  Multiple complication score; number of observations – creatinine/urea (MM = 11, SM-S = 9, CM-S = 38, CM-NS = 18); bilirubin in adults (MM = 17, SM-S = 11, CM-S = 43, CM-NS = 22); TLC (MM = 46, SM-S = 20, CM-S = 68, CM-NS = 18); *p* values of numeric variables shown in bold were calculated by ANOVA and others were by Kruskal-Wallis rank test. * *p* value excluding MM group; **p value = 0.0082 (kruskal-wallis) between CM-S and CM-NS. Outcome of 8 cerebral malaria cases and 2 severe malaria cases is unknown and thus have not been included in analysis. IQR  =  Inter-quartile range. N/A  =  Not applicable.

Respiratory rate in SM-S (*p* = 0.0001), CM-S (*p* = 0.0001) and non survivor group (CM-NS + SM-NS) was significantly higher (*p*<0.0001) than MM patients. Systolic blood pressure was significantly lower in SM-S (*p* = 0.0001) and higher in non survivor group (*p* = 0.0003) than MM but it was statistically insignificant between CM-S and MM patients (*p* = 0.945). Percent peripheral parasitemia was significantly higher in SM-S (*p* = 0.0001), CMS (*p* = 0.0034) and non survivors patients (*p* = 0.0003) than MM patients.

Pulse rate was also significantly higher in SM-S (*p* = 0.0071), CM-S (*p* = 0.035) and non survivors (*p* = 0.0007) compared to MM patients. Hemoglobin level was significantly lower in SM-S (*p* = 0.0001), CM-S (*p* = 0.0003) and non survivors (*p* = 0.0005) groups compared to MM. Serum bilirubin levels were significantly higher in SM-S, CM-S, and non survivors compared to MM (*p* = 0.0001 for all). Blood urea was also significantly higher in CM-S (*p* = 0.039) and non-survivors (*p* = 0.014) compared to MM.

The multiple complication score was significantly higher in non survivors group compared to SM-S (*p* = 0.0001) and CM-S (*p* = 0.277). Patients with 2 complications (*p* = 0.0011) and those with ≥3 complications (*p* = 0.0001) had significantly higher median percent parasitemia than patients with single complication. No significant difference was found between patients with 2 and ≥3 complications. Hospital stay period of SM-S patients (p = 0.018) and CM-S patients (p = 0.0001) was significantly longer than MM cases. Complicated malaria patients with ≥3 complications also had significantly longer hospital stay than those with single complication (*p* = 0.05) but not double complications (*p = *0.448).

### Demographic and background variables

Univariate logistic regression analysis revealed that illiteracy, work profile as unskilled labourer among adults and subject's belonging to an ethnic tribe group were the significant risk factors for complicated malaria (Table 3). However, multivariate analysis revealed that only work profile and ethnicity were found to be independently associated with risk of complicated malaria. On the other hand ethnicity was the only one variable associated with mortality in univariate and multivariate analysis. Though complicated malaria was found more in ethnic tribes, mortality among them was comparatively less than non tribes (Table 4). Mortality in complicated malaria among some of the major ethnic tribal groups noted in the present study was 30.2% in Batra tribes (13 out of 43 cases), 18.8% in Maria tribes (3 out of 16 cases), 15.4% in Dhruva tribes (4 out of 28 cases) and 8.3% in Muria tribes (1 out of 12 cases).

**Table 3:** Univariate and multivariate logistic regression analysis of different background variables associated with complicated malaria at Maharani Hospital Jagdalpur, Chhattisgarh, India.

Background variables	Numerator/Denominator (%)	Odds ratios (95%CI)	Adjusted odds ratios (95%CI)
Literacy (adults)		2.0 (1.2–3.3), P = 0.011	1.0 (0.5 – 2.2), P = 0.936
(ref) Literate	105/187 (56.2)		
Illiterate	73/102 (71.6)		
Work Profile (adults)		3.6 (2.0–6.5), P <0.0001	3.0 (1.5 – 5.9), P = 0.002
(ref) Semiskilled/Skilled	40/84 (47.6)		
Unskilled	95/124 (76.6)		
Sleeping habit		0.7 (0.3 – 1.4), P = 0.316	N/A
(ref) Indoor	207/312 (66.3)		
Outdoor	19/33 (57.6)		
Use of mosquito protection methods		0.9 (0.6 – 1.5), P = 0.717	N/A
(ref) Yes	159/239 (66.5)		
No	71/110 (64.5)		
Tribe		2.4 (1.5–3.9), P <0.0001	1.9 (1.0 – 3.6), P = 0.047
(ref) No	66/125 (52.8)		
Yes	164/224 (73.2)		

Complicated malaria (n = 232) was coded as 1 and mild malaria (n = 119) as 0. Similarly two groups of an independent variable listed above were also coded as “0” and “1”. Ref  =  Reference for odds ratios; CI  =  Confidence interval; N/A  =  Not applicable. Variables which were significant (*p*∼0.1) in univariate analysis were taken in multivariate analysis). Complicated malaria included 176 cerebral malaria and 56 severe malaria (non cerebral) cases.

**Table 4:** Univariate and multivariate logistic regression analysis of different demographic and background variables associated with prognosis of enrolled complicated malaria patients admitted at Maharani Hospital Jagdalpur, Chhattisgarh, India.

Background variables	Numerator/Denominator (%)	Odds ratios (95%CI)	Adjusted odds ratios (95%CI)
Literacy (adults)		1.3 (0.7–2.5), P = 0.446	N/A
(ref) Literate	28/102 (27.5)		
Illiterate	23/70 (32.9)		
Work Profile (adults)		0.7 (0.3–1.6), P = 0.403	N/A
(ref) Semiskilled/Skilled	13/38 (34.2)		
Unskilled	25/93 (26.9)		
Sleeping habit		0.3 (0.1–1.3), P = 0.111	0.2 (0.1–1.1), P = 0.070
(ref) Indoor	56/197 (28.4)		
Outdoor	2/19 (10.5)		
Use of mosquito protection methods		0.8 (0.4–1.6), P = 0.509	N/A
(ref) Yes	44/154 (28.6)		
No	16/66 (24.2)		
Tribe		0.4 (0.2–0.8), P = 0.007	0.4 (0.2–0.8), P = 0.006
(ref) No	26/63 (41.3)		
Yes	36/157 (22.9)		

Fatal form of complicated malaria (n = 62) was coded as 1 and non fatal complicated malaria (n = 160) as 0. Similarly two groups of an independent variable were also coded as “0” and “1”. The outcome of 8 cerebral malaria (CM) and 2 severe malaria (SM) patients is unknown. Ref  =  Reference for odds ratios; CI  =  Confidence interval; N/A  =  Not applicable. Variables which were significant in univariate analysis (*p*∼0.1) were taken in multivariate analysis). Complicated malaria included 168 CM and 54 SM (non cerebral) cases with known outcomes.

### Clinical pattern of complicated malaria among adults and children

Analysis of complication patterns of adults (n = 182) and children (n = 50) with complicated malaria revealed that the frequency of seizures, hyperparasitemia, severe malaria anemia, hypoglycemia, respiratory distress, and blood transfusion any time during hospitalization were significantly associated with children than adults. Where as complications like jaundice and acute renal failure were significantly associated with adults than children (Table 5). Coma, abnormal bleeding/hemolysis and shock were statistically insignificant between adults and children.

**Table 5:** Clinical comparison between enrolled adults and children diagnosed with complicated malaria (N = 232) at Maharani Hospital Jagdalpur, Chhattisgarh, India.

Factors/Complications	Adults (N = 182)	Children (N = 50)	χ^2^, *p* value
	Numerator/Denominator (%)	Numerator/Denominator (%)	
Coma	135/182 (74.2)	41/50 (82.0)	1.3, 0.252
Seizure	57/180 (31.7)	39/49 (79.6)	36.3, <0.00001
Hyperparasitemia	57/176 (32.4)	25/47 (53.2)	6.9, 0.0086
Jaundice	67/150 (44.7)	1/32 (3.1)	19.5, <0.00001
Hemolysis	19/164 (11.6)	9/44 (20.5)	2.3, 0.125
Respiratory distress	43/170 (25.3)	19/46 (41.3)	4.5, 0.033
Renal failure	36/172 (20.9)	3/48 (6.3)	<0.00001
Shock	28/174 (16.1)	10/42 (23.8)	1.4, 0.238
Severe malaria anemia	17/182 (9.3)	10/50 (20.0)	4.3, 0.037
Blood transfusion	17/173 (9.8)	15/46 (32.6)	15.1, <0.0001
Hypoglycemia	4/182 (2.2)	6/50 (12.0)	0.0079
Multiple complications	127/158 (80.4)	38/44 (86.4)	0.8, 0.364

Categorical variables were compared by chi square/fisher exact test as appropriate and significance is calculated for children with respect to adults (reference).

Complicated malaria included 176 cerebral malaria and 56 severe malaria (non cerebral) cases.

### Logistic regression analysis and prognosticators

Univariate logistic regression analysis for prognostic indicators revealed that coma, jaundice, hemolysis/abnormal bleeding, respiratory distress, acute renal failure and multiple complications were significantly associated with mortality of severely ill patients (Table 6). We found that, seizure, hyperparasitemia, hypotension, severe malaria anemia and hypoglycemia were not associated with mortality risk. In multivariate analysis only coma was found to be significantly associated with mortality outcome.

**Table 6:** Univariate and multivariate logistic regression analysis of different clinical complications associated with prognosis of enrolled complicated malaria (CM+SM) patients admitted at Maharani Hospital Jagdalpur, Chhattisgarh, India.

Complications	Numerator/Denominator (%)	Odds ratio (95%CI)	Adjusted odds ratio (95%CI)
Coma		5.0 (1.9–13.3), P = 0.001	3.8 (1.04–13.7), P = 0.043
Yes	57/168 (33.9)		
(ref) No	5/54 (9.3)		
Seizures		1.2 (0.6–2.2), P = 0.587	N/A
Yes	26/90 (28.9)		
(ref) No	33/129 (25.6)		
Hyperparasitemia		1.0 (0.5–1.9), P = 0.987	N/A
Yes	22/77 (28.6)		
(ref) No	39/137 (28.5)		
Jaundice		2.0 (1.0–3.9), P = 0.051	1.4 (0.6–3.4), P = 0.405
Yes	23/66 (34.8)		
(ref) No	23/108 (21.3)		
Hemolysis/abnormal bleeding		2.9 (1.3–6.6), P = 0.011	2.2 (0.8–6.0), P = 0.117
Yes	13/28 (46.4)		
(ref) No	39/170 (22.9)		
Respiratory distress		2.8 (1.5–5.4), P = 0.002	2.3 (1.0–5.2), P = 0.058
Yes	26/59 (44.1)		
(ref) No	32/147 (21.8)		
Acute renal failure		2.7 (1.3–5.7), P = 0.01	1.4 (0.5–3.7), P = 0.560
Yes	16/36 (44.4)		
(ref) No	40/174 (23.0)		
Hypotension		0.7 (0.3–1.6), P = 0.366	N/A
Yes	8/37 (21.6)		
(ref) No	49/169 (29.0)		
Severe malaria anemia		1.7 (0.7–4.1), P = 0.207	N/A
Yes	10/26 (38.5)		
(ref) No	52/196 (26.5)		
Hypoglycemia		1.8 (0.5 – 6.5), P = 0.390	N/A
Yes	4/10 (40.0)		
(ref) No	58/212 (27.4)		
Multiple complications		8.5 (2.0–36.6), P = 0.004	N/A^*^
Yes	55/159 (34.6)		
(ref) No	2/34(5.9)		

CM: Cerebral malaria; SM: Severe malaria; Fatal complicated malaria was coded as 1 (n = 62) and non fatal complicated malaria as 0 (n = 160). All the listed independent variables were also coded as 1(yes) and 0(no). Note: Outcome is not known in 8 CM cases and 2 SM cases; CI  =  Confidence interval; N/A  =  Not applicable. Variables which were significant (*p*∼0.1) in univariate analysis were not taken in multivariate analysis; *Multiple complications were considered as derived variable hence has not been considered for multivariate analysis. Complicated malaria included cerebral malaria and severe malaria (non cerebral) cases.

### Multiple complications in complicated malaria

Patients with two complications (71 cases, 19 deaths) and ≥3 complications (94 cases, 36 deaths) had significantly higher mortality than patients with single complication (n = 36, 2 death). Odds of mortality also increased in patients with two complications [6.0, 95%CI (1.2–29.3), *p* = 0.0114] and ≥3 complications [10.1, 95%CI (2.1–49.3), *p* = 0.0004] compared to single complication. Mortality was significantly associated with multi-organ complication score (r = 0.26, *p* <0.0003).

### Complicated malaria and mixed infection

We recorded a total of 11 microscopically positive mixed infections (*Pf* + *Pv*) (0.5% of 2110 cases associated with *P. falciparum* malaria). Of these eleven cases, three had SM (27.3%) of which two died (18.2%). Additionally PCR revealed 14 mixed infections (10 *Pf* + *Pv,* 2 *Pf* +*Pv*+*Po*, 2 *Pf*+*Po*), of which mortality was recorded in 28.6% (4 cases).

### Recovery and mortality patterns of severe cases

**Figure 3:**
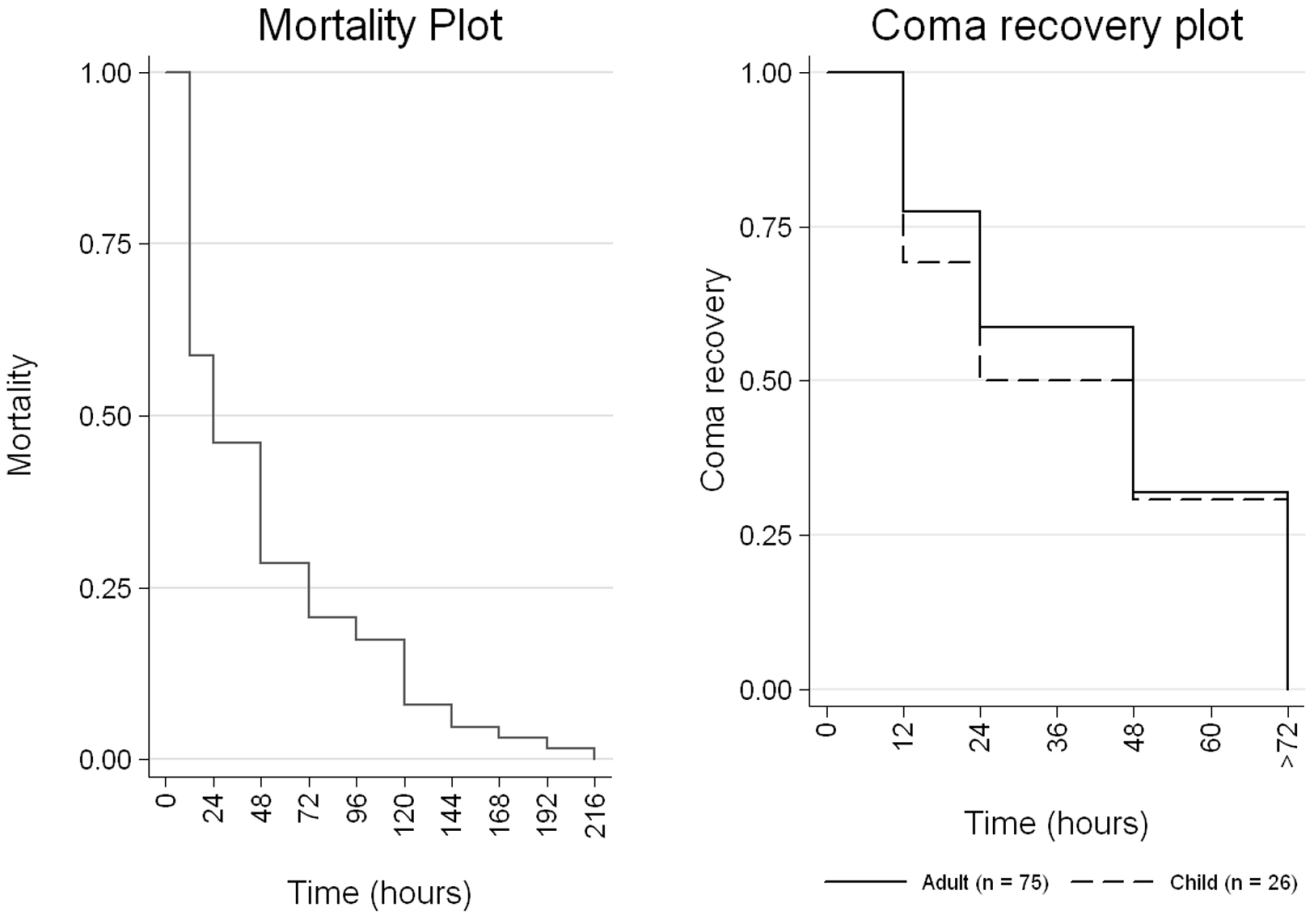
Time period wise coma recovery and mortality pattern of *P. falciparum* associated complicated malaria cases in a tertiary care hospital in Bastar, Chhattisgarh, Central India.

Seizures during hospitalized period were recorded in 29 cases (3 SM and 26 CM) majority of them were within 12 hours of admission (62.0%) and associated with 34.5% mortality (all CM). Out of 176 analyzed CM cases (57 died), coma patterns of recovered patients were available for 101 cases (75 adults and 26 children). Children showed relatively faster recovery than adults but it was statistically insignificant at all the time intervals for first 48 hours (Fig. 3). Of 101 subjects, 24.8% showed very fast recovery from coma i.e., within 12 hours (30.8% children and 22.7% adults). There was no difference in coma recovery pattern between males and females among adults as well as children.

Time point wise mortality pattern (adults  = 52, children =  11) revealed that 54.8% mortality occurred between admissions to 24 hours (Fig. 3). Among adults 75% (9/12) females and 32.5% (13/40) males died within 12 hours of admission (χ^2^ = 6.8, P = 0.009). Children also showed similar trends.

### Outcome of referred cases

Out of the 232 enrolled complicated malaria, seventeen cases were referred to specialty care centre in Raipur of which 29.4% died. All had CM and other associated organ dysfunctions (94%).

## Discussion

Assessment and analysis of the local malaria situation are a prerequisite for embarking on any control and elimination program. This study was conducted to determine the clinical features in patients with complicated malaria among the predominantly tribal population. Differences in age wise pattern of severe and complicated malaria (peak observed among children>4–5 years) were noticed compared to the presentation of malaria in various other endemic settings of India and Africa [3], [4], [5], [6], [7] [22].

Age wise pattern of malaria and associated clinical manifestations were found, which differ from prior reports from this part of the Central India. In this study the median age of children having malaria and associated morbidity was 5 years. In Cuttack, the median age of severe malaria in children was 8 years [5], in contrast to the mean age of about 2 years in high transmission areas in Africa [22]. In lower transmission areas of Africa the mean age seems to be higher (6.2 years) [23]. Further, in Cuttack, mortality in children (12%) was almost equally distributed among different age groups with the exception of a slightly higher rate (16%) in children <2 years. In this study highest CFR was found in children aged>4–5 years with a mean age of mortality 7.5 years. In high transmission areas of Africa, mortality seems to be very rare in patients>4 years old [22]. However, a similar mean age of death at 7.5 years has been found in non-endemic regions of Africa [23].

A review of the age pattern of malaria revealed that as transmission increased there was a shift of malaria towards younger age groups regardless of seasonality [24], [25]. This depends on the age, intensity of transmission and immunity of the patients [3]. More deaths among young children suggest that they are more vulnerable and needed special care during management. Thus, defining the clinical spectrum of malaria in different age group is necessary for studies conducted in areas of different malaria endemicity and transmission dynamics.

Further, in this study 14.0% cases had CM that was not associated with any other complication compared to 8% reported earlier [26]. Gender wise differences in malaria morbidity have been recently described in a hospital based surveillance study [27]. Observations in the present study were similar as among adults (not in children), males showed significantly higher malaria and *P. falciparum* than females. However, the proportions of severity and mortality in *P. falciparum* malaria were independent of gender difference in adults or children. Treatment seeking behaviour, occupational activities and socio-cultural barriers may also cause differences towards treatment at higher centres between males and females [28], [29].

Among prognostic indicators respiratory distress, coma, multiorgan dysfunction and hyperparasitemia have already been described to be independently associated with mortality among Indian children earlier [5], whereas among Indian adult jaundice, renal failure and multiple complications were the predictors of mortality [3]. We have found that the coma was the only independent predictors of mortality in all age groups. Since multi-organ complication is a derived variable we have not considered it as an independent variable.

Further, there are reports in literature providing evidence of increasing severity and morbidity in patients of mixed infections [30], [31]. In this study also mixed infections were more common in complicated cases than mild form of the disease. Therefore, multicentric clinico-epidemiological studies are needed to provide evidence for further research on the pathogenic potential of mixed infections. The knowledge of mixed infection is important not only for developing appropriate control measures but also for therapeutic options.

In most of the previous studies complicated malaria mortality is reported to occur within first 24 hours of admission before the full dose of effective antimalarial is administered [3], [32], [33]. We also have recorded nearly 50% mortality within 24 hours of admission. Host's immunity, misdiagnosis or late reporting, and delayed treatment may be the cause of early mortality [7], [34]. In children pathogenesis is very quick in the absence of timely treatment and some proportions of children might die even before reaching health care facility. Prior studies have shown that CM associated mortality ranged from 14% – 23% in India [4], [7], [34]. CM/SM associated mortality in the present study was 32.4% and 8.9% respectively among PCR confirmed enrolled cases.

From the clinical perspective, this study has some limitations also. We could not collect data on neurological sequelae at the time of discharge of complicated malaria patients in this study. Hypoglycemia was also the limitation of this study as blood glucose could not be measured before intravenous infusion, which invariably was started immediately in the casualty unit after first clinical examination in severe cases. Upon clinical examinations, as indicated, biochemical investigations were advised by the consultant physician but this information was not available for all the cases. Hemoglobinopathy (sickling and G6PD deficiency) data was also not available. Further, it is possible that we did not capture the full burden of malaria and the complete spread of clinical symptoms in our hospital setting. It is also possible that many deaths take place at home in the villages and there is a possibility that we missed certain manifestation of malaria causing early deaths in children.

## Conclusion

Severe and complicated malaria is a challenge to the attending physician who is treating an admitted severe malaria patient at a remote health facility. This comprehensive prospective study showed that delayed referral and delay in starting parenteral therapy could be an important factor for poor outcome. To reduce malaria related mortality provision has to be made to refer the cases of complicated malaria after giving initial treatment from peripheral centre or from home by health workers. Relatively high morbidity and mortality among young children suggest that they are more vulnerable and needed special care during management. This study showed that there is need of site specific data in order to understand the clinical and epidemiological picture for developing appropriate intervention strategy and management guidelines.
